# Olfactory-Related Quality of Life in Multiple Chemical Sensitivity: A Genetic-Acquired Factors Model

**DOI:** 10.3390/ijms21010156

**Published:** 2019-12-25

**Authors:** Alessandro Micarelli, Andrea Cormano, Daniela Caccamo, Marco Alessandrini

**Affiliations:** 1 Institute of Mountain Emergency Medicine, EURAC Research, I-39100 Bolzano, Italy; 2 ITER Center for Balance and Rehabilitation Research (ICBRR), 02032 Rome, Italy; 3 Domus Medica, Bagnoli del Trigno, 86091 Isernia, Italy; cormano_andrea@libero.it; 4 Department of Biomedical Sciences, Dental Sciences and Morpho-functional Imaging, Polyclinic Hospital University, 98124 Messina, Italy; dcaccamo@unime.it; 5 Department of Clinical Sciences and Translational Medicine, University of Rome “Tor Vergata”, 00133 Rome, Italy; malessandrini63@gmail.com

**Keywords:** multiple chemical sensitivity, olfactory disorder, genotype analysis, quality of life, environmental exposure, xenobiotics detoxification

## Abstract

Genetic polymorphisms as well as environmental exposures to chemical compounds, iatrogenic, psychological, and physical trauma may play a pathophysiological role in multiple chemical sensitivity (MCS) olfactory complaints, given that xenobiotic metabolism is influenced by sequence variations in genes of metabolizing enzymes. Thus, the aim of the present study was to depict—by means of multiple regression analysis—how different genetic conditions, grouped according to their function as well as clinical background and environmental exposure may interfere with those olfactory complaints referred by MCS patients. Therefore, MCS patients after gene polymorphism sequencing, the olfactory-related quality of life score—calculated by means of the Questionnaire of Olfactory Disorder in forty-six MCS patients—have been found to significantly rely on the phase I and II enzymes score and exposure to previous compounds and surgical treatments. The present work—implementing for the first time a genetic-acquired factors model on a regression analysis—further reinforces those theories, positing MCS as a complex, multifactorial, disease in which the genetic risk related to phase I and II enzymes involved in xenobiotic detoxification, olfactory, and neurodegenerative diseases play a necessary, but probably not sufficient role, along the pathophysiological route of the disease.

## 1. Introduction

Multiple chemical sensitivity (MCS) is a relatively common clinical diagnosis in Western populations [1]. The prevalence of self-reported chemical sensitivity symptoms in population-based studies ranges from 9 to 33% [2], whereas physician-diagnosed MCS or reports of disabling consequences in the form of social and occupational disruptions are much lower, ranging from 0.5 to 6.3% [2,3].

MCS patients usually react to a wide range of everyday chemical compounds such as petrol, perfume, or pesticides by complaining of a wide spectrum of symptoms ranging from headache, fatigue, respiratory symptoms, dizziness, nausea, and especially, disosmia [4,5,6]. The discussion on the definition and nomenclature reflects the fact that the etiology of MCS is still unclear and a matter of debate [4,6].

Most frequently discussed etiologies include neurogenic inflammation [7], classical conditioning [8], and biochemical disruptions [1,6,9], finally hypothesizing that environmental exposure to chemical compounds, iatrogenic, psychological, and physical trauma as well as genetic polymorphisms may play a pathophysiological, mutually fostering role in MCS given that xenobiotic metabolism is influenced by sequence variations in the genes of metabolizing enzymes [6,10]. This is particularly evident—beyond controversial literature among olfactory testing results [1,5,11,12,13,14,15]—when focusing on those daily activities negatively impacted by MCS olfactory disperception, which finally accounts for higher scores demonstrated by these patients along the Questionnaire of Olfactory Disorders (QOD), representing one of the most frequent clinical complaints referred by MCS patients [1,12,13]. Such debate on the mechanisms relating olfaction and pathophysiological processes at the central level in MCS reflects uncertainties among those phenomena regarding the pathways through which environmental compounds may elicit responses from sensorial organs to the central nervous system. In fact, if on one hand, small airborne molecules may interact with the olfactory epithelium [16], on the other hand, non-volatile substances such as steroids [17], peptides [18,19], and proteins [20] can be detected only by the vomeronasal organ (VNO) [21], a partially atrophic, even if functional in many subjects, organ that has been suspected to be the primary target of olfactory steroids [22]. In this light, the expression of sex hormone binding globulin, a transport protein for estradiol and testosterone, and vitamin D binding protein and receptor in the VNO has been recently observed [23,24,25]. Furthermore, biosynthesis and metabolism of these neuroactive steroids also occur within the brain [26,27] where they are involved in limbic functions [28,29]. These aspects have led to the proposal that a sensory–cognitive–physiologic pathway mediated by the VNO, or due to a direct effect for receptor co-localization in the brain, may also exist, thus representing an alternative candidate mechanism for the phenomenon of MCS beyond the olfactory-mediated responses to low levels of airborne chemicals [30].

This burden is of additional particular relevance in MCS cases where the absence of a dose–response relationship and of a characteristic symptom pattern may be identified, thus leading to no reliable physiological markers that can be used to separate sufferers from non-ill individuals nor to pathomechanisms that can be clearly advocated to explain the symptoms, especially those affecting the sense of smell and their daily consequences [31,32,33,34].

Consequently, MCS patients are offered insufficient healthcare solutions and experience being met with doubt or limited understanding of their condition by healthcare professionals, the social welfare system, and society in general [35,36,37]. It is therefore essential that a deeper knowledge about the pathogenic pathways leading to symptoms elicitation in MCS participants is being generated [38].

In this vision, many attempts—with different results—have been conducted in order to highlight the possible relationships between genetic polymorphisms and quality of life tests and to depict possible routes of pathophysiological models underpinning the development of MCS-related complaints. Among the vast literature regarding the gene polymorphisms involved in MCS, the overall approach is, at least, to define the individual genetic profiles of enzymes involved in body detoxification from xenobiotics such as phase I metabolism cytochrome P450 monoxygenases (CYP450), phase II metabolism glutathione-S-transferase (GST) and N-acetyl-transferase (NAT2), antioxidant defense, namely mitochondrial superoxide dismutase (SOD2), paraoxonase 1 and 2 (PON1, PON2), endothelial and inducible nitric oxide synthase (NOS2, NOS3) as well as folate cycle/methylation (MTHFR) [10,31,39,40,41,42,43,44,45,46]. The hypothesis underlying the above cited investigations was the fact that the extreme individual sensitivity in MCS patients could be related to the inherited impairment in xenobiotics/endobiotics metabolism and to a vicious cycle [47] including peroxynitrite overproduction and lipid peroxidation, possibly not coped by the impairment in SOD, catalase, glutathione peroxidase, and NOS enzyme activities [48].

Thus, given (i) the fact that the more endorsed models underpinning the MCS pathomechanisms rely on the reciprocal influences between environmental exposure, clinical background, and the genetic profile involved in xenobiotics metabolism, and ii) the lack of literature regarding the possible impact of this multifactorial model on olfactory behavior, the aim of the present study was to depict, by means of multiple regression analysis, how different genetic conditions grouped in light of their function as well as clinical background and environmental exposure may interfere with those olfactory complaints referred by MCS patients.

## 2. Results

Forty-nine consecutive MCS patients were enrolled. Among them, one was using antidepressant drugs, one reported a history of alcohol abuse, one of diabetes, and were therefore excluded. Therefore, 46 MCS patients (27 women and 19 men; mean age 47.2 ± 10 years) met the eligible criteria and were included in the study. Table 1 depicts the clinical–anamnestic data of the MCS population in the study and the number and percentage of patients for each class of enzymes and for the other factors computed in the regression model as well as their score average.

The analysis of polymorphisms in genes coding for enzymes involved in xenobiotic metabolism pathways showed that the frequencies of CYP2C9*2 and *3 polymorphisms, in heterozygous state and double heterozygous state, were significantly higher in MCS patients compared with those in the Caucasian general population available from the literature (Table 2). Individuals bearing either the *1/*2 or the *1/*3 genotype were classified as CYP2C9 poor metabolizers, while those with the *2/*3 diplotype were considered very poor metabolizers. Although a higher frequency was observed for CYP2C19 *1/*2 and *1/*17 genotypes, associated with the phenotype poor metabolizer (PM), no significant differences resulted when comparing MCS patients with the Caucasian general population, likely due to the small number of recruited subjects.

The following CYP2D6 alleles were included in this study with functional status as assigned: normal enzyme activity (functional) *1, *2, *2xN, *2A; reduced enzyme activity: *10, *41; null enzyme activity: *4, *6 [49]. The frequencies of CYP2D6 mutated alleles were similar to those described in the Caucasian general population [50]. Individual phenotypes were inferred from participants on the basis of the commonly assigned function for allele combinations, as follows: functional/functional (extensive metabolizer, EM), functional/reduced (EM), functional/null (EM), reduced/reduced (intermediate metabolizer, IM), reduced/null (IM), null/null (poor metabolizer, PM). The frequencies of the called phenotypes were: PM = 6.5%, IM = 6.5%, EM = 80.5%, and UM = 6.5%. The proportion of these phenotypes was within the range estimated in a general Caucasian population by Crews et al. [50]. The majority of the EM phenotype group possessed the CYP2D6 *1/*1 genotype (28.3%), followed by people having the CYP2D6 *2/*2, and *2A/*2A. Significant differences were found only after comparison of the IM MCS patients with IM in the Caucasian general population (Table 2).

A higher prevalence was found for mutated alleles of GST isoforms, namely GSTP1 313G, GSTM1 Del, and GSTT1 Del as well as for the variant UGT1A1*28 in MCS patients compared with the Caucasian general population. However, these differences did not reach statistical significance, or only in some cases tended to statistical significance, likely due to the small number of recruited subjects.

As a whole, these findings indicate that MCS patients have an impaired metabolism of xenobiotics, both in phase I and phase of body detoxification, and confirm previously reported observations [40,43,44,51,52].

When compared with the Caucasian general population, MCS patients presented with significantly higher frequencies of polymorphisms in genes coding for antioxidant enzymes, namely SOD2 A16V, CAT -C262T, and PON1 L55M (Table 1). Gene polymorphisms examined in our study are known to greatly affect antioxidant enzyme activities due to either resulting amino acid substitutions or the effects on gene transcription rate [53,54,55,56]. The resulting individual phenotype is characterized by a reduced antioxidant defense, potentially leading to increased susceptibility to oxidative stress. The observed increased prevalence of defects in antioxidant enzymes confirms previous results [10,40,51,52] and may provide a mechanistic explanation for reported MCS features of oxidative stress [48].

No significant differences were observed for the distribution of polymorphisms in genes coding for enzymes involved in DNA methylation and repair pathways, namely MTHFR and DNA repair enzyme 8-oxoguanine glycosylase 1 (OGG1) as well as for nitrosative stress-related enzyme NOS3 and immune response enzyme myeloperoxidase (MPO) (Table 2). The increased frequencies of NOS3 TT894, MTHFR TT677, and MTHFR AC1298 in MCS patients compared with the Caucasian general population are in agreement with previously published observations [40,45,51,57].

Multiple regression analysis was run in order to determine the results of LQrv in relation to the nine prognostic factors. The correlation was statistically significant only for the phase II class score, phase I class score, chemical compounds exposure, and previous surgery with partial correlation coefficient of 0.23, 0.28, 0.3, and 0.2, respectively (Table 3).

**Figure 1:**
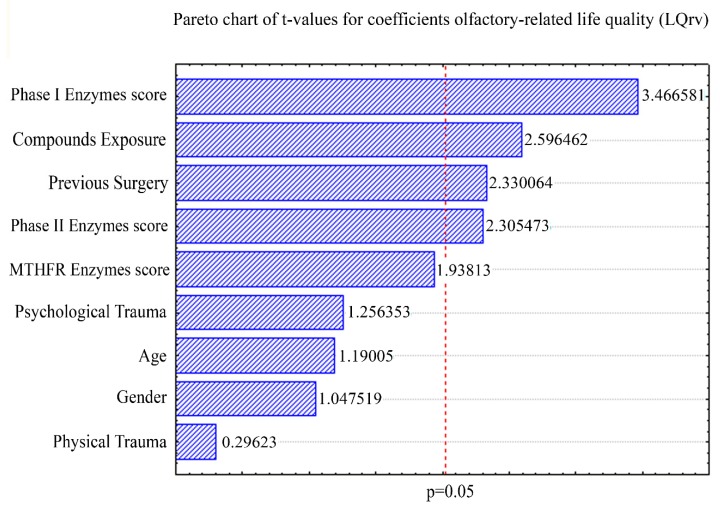
Pareto chart of t-values for all coefficients in the regression models. Pareto chart contrasting t-values of all coefficients accounting for the regression model with the significant p-value cut-off (vertical dotted red line).

The multiple regression Equation (1) is as follows:

(1) $$\begin{array}{ll} X=0.87996_{x1} & +0.40192_{x2}+1.09827_{x3}+2.53639_{x4}\\ & +1.25605_{x5}-0.28221_{x6}+1.89423_{x7}+0.04117_{x8}\\ & +0.81441_{x9}+18.63568 \end{array}$$

where *X* is the predicted value of the LQrv; *x*_1_ is the MTHFR class score; *x*_2_ is the phase II class score; *x*_3_ is the phase I class score; *x*_4_ is the previous event of chemical compounds exposure (0 for absence and 1 for presence); *x*_5_ and *x*_6_ are the previous events of psychological and physical trauma (0 for absence and 1 for presence), respectively; *x*_7_ is the event of previous surgery (0 for absence and 1 for presence); *x*_8_ is the patient’s age and *x*_9_ is the patient’s gender (1 for female and 0 for male) (Table 3) for which t-values were contrasted on the significant *p*-value cut-off on a Pareto chart (Figure 1). The multiple correlation coefficient was 0.88 with a *p* value less than 10^−4^.

**Figure 2:**
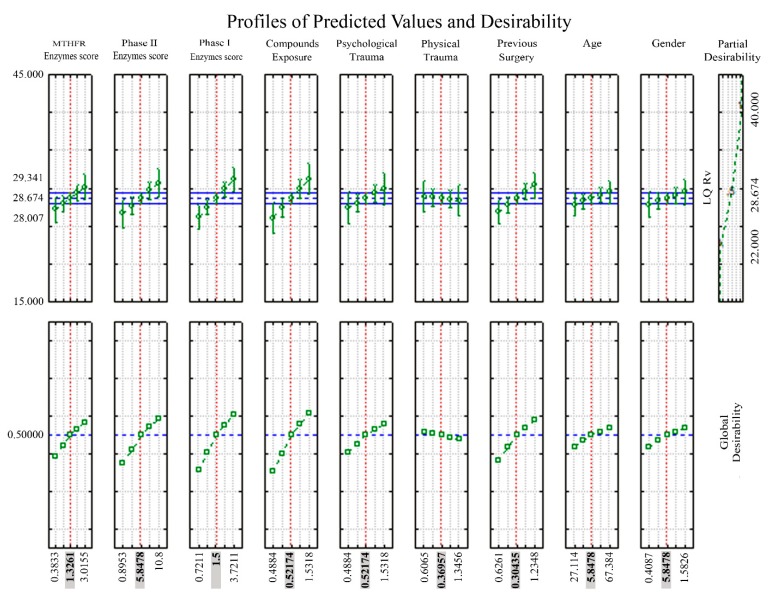
Profiles of predicted values for all coefficients in the desirability model. Plot depicting in MCS subjects the predicted values (bottom squares, grey highlighted) of the main prognostic factors when contrasted against the range included within the upper and lower limits of olfactory-related quality of life score (LQrv) (top squares) in the desirability model.

Finally, Table 4 and Figure 2 depict the desirability model results in partial and global desirability values for each prognostic factor, particularly depicting a cut-off value of the phase I class score, phase II class score, previous events of chemical compounds exposure, and surgery equal to 1.5, 5.84, 0.52, and 0.3, respectively in order to obtain a LQrv score at least equal to 28.67.

## 3. Discussion

The first interesting aspect of the present study resides in those partial coefficients depicting a multifactorial model contributing to one of the most complained symptoms in MCS, in other words, olfactory alterations which beyond a wide sensorial discomfort referred in this disorder [1,6,12,13,68,69,70] have been found to severely impact on the quality of life and routine activities of MCS patients [1,6,12,34]. In particular, when paying attention to both the regression model and the Pareto chart highlighting respective t-values accounting for significant impact on LQrv, such a multifactorial model was hierarchically contributed to by genetic factors (phase I and phase II classes scores), environmental (chemical compound exposures), and anamnestic characteristics (presence of previous surgery events) of the patients. This tends to confirm previous hypotheses suggesting the inherited and acquired dysfunction of the chemical defensive system as a molecular basis for MCS complaints [42,48]. Indeed, adequate body response to environmental toxicants presumably requires proper function of the xenobiotic detoxification pathways. Among those factors contributing to variability in human response to toxicants, it can be expected that inherited and acquired variations in the metabolism and excretion of xenobiotics play a major influence. Indeed, it is well known that fat soluble xenobiotics are typically absorbed from the digestive tract, oxidized to intermediates that may be highly reactive, conjugated to increase their solubility, then excreted either by the kidneys in urine, or by the liver in bile [71]. Specifically, elimination of renally conserved, nonpolar fat soluble compounds tends to be more troublesome to the human body and requires sequential metabolic steps. In particular, phase I metabolism of xenobiotics typically activates nonpolar toxic compounds to make them more reactive through oxidation mediated by the cytochrome P450 family of enzymes. On the other hand, phase II conjugation reactions take compounds with an active functional group including compounds activated in Phase I reactions and add an endogenous substrate to that group to make the compounds more soluble and/or reduce their toxicity. Phase II conjugates include glucuronic acid, sulfate, glutathione, acetyl, glycine, and methyl groups [71,72,73]. According to the general trend in literature, oxidative stress and diminished capacity in detoxifying mechanisms have been involved in the pathophysiology of MCS [9]. In particular, the pivotal point in the genesis of the MCS seems to reside in the strict connection existing between oxidative stress damage, neurogenic inflammation, and neural disorders [74,75], based on the vulnerability of the central nervous system to free radicals. In light of this, the present results further strengthen those studies [76,77], highlighting that neurogenic inflammation could be multi-factorially underpinned by genetic predisposition to the breakdown of oxidative stress mechanisms and exposure to environmental events. Following these assumptions, once neurogenic inflammation, together with central biochemical processes [9,78], has been established, according to the neural sensitization theory, MCS could be attributed to a pathological hyper-reactivity of neurons in some areas of the brain, mainly in the olfactory and limbic systems [79], inducing an over-reactivity to external stimuli in different end-organs [6], possibly relying on a olfactory-limbic kindling model [6,79].

Interestingly, the same pathways involved in the metabolism of xenobiotics have been previously linked both to impairment in the olfactory system and involvement in neurodegenerative as well as psychiatric diseases [80]. Previous studies focusing on MCS patients demonstrated altered olfactory sensitivity [1,13,34], although a direct correlation between detoxification pathways, chemical exposures, and/or anamnestic events has not been definitively established, possibly due to weaknesses related to MCS screening, relatively low accuracy of objective olfactory testing, and the absence of correlation between factors and specific tests investigating the quality of life in relation to olfactory disorders. However, many hypotheses have postulated that the chronic exposure (low-dose, overtime) of biogenic amines-based-pesticides (neonicotinoids and formamidines) may disrupt neuronal cholinergic and octopaminergic signaling and produce excessive reactive oxygen species and reactive nitrogen species [9,47,78,81]. These, in turn, may react with macromolecules and interfere with the mitochondrial respiratory chain and mitochondrial Ca2+ metabolism [9,47,78,81]. Oxidative stress has proven to impair cognitive behavior including olfactory learning and memory, especially in those conditions where detoxification pathways may not properly counterbalance the generation of damage [81,82]. This appears to be of much interest, considering that the effects of odors and chemical compounds are not only exerted by the stimulation of the olfactory system through inhalation, but they can also enter the body through absorption in the skin, nose, and mouth, and thereafter, enter the blood stream, thus reaching the brain due to their lipophilic characteristics and causing broad spectrum types of effects [83] such as breakdown in the subjective experience of odor [84].

We here demonstrated significant differences in the distribution of gene polymorphisms of enzymes involved in xenobiotic metabolism, oxidative stress, and DNA methylation/repair pathways between the MCS cohort here studied and the Caucasian general population, with a higher prevalence of gene defects in MCS patients than in healthy subjects (Table 2). Following the above-mentioned hypothesis that postulates the overlap between breakdown of detoxification pathways, neuropsychiatric disorders, and olfactory dysfunction, it is not surprising that the multiple regression and the desirability model demonstrated that phase I and II scores were increasingly associated with the risk of neuropsychological and quality of life consequences of the olfactory dysfunction in MCS (Table 3, Figure 1). By way of example, GSTs, accounting for phase II class score, are a family of multifunctional enzymes playing a central role in the detoxification of toxic and carcinogenic electrophiles [85], extensively indagated in MCS [52]. In fact, GST isoenzymes catalyze the conjugation of glutathione to a variety of electrophilic compounds including formaldehyde. If the lack of GSTM1 activity, which detoxifies the reactive metabolites of benzo[a]pyrene and other polycyclic aromatic hydrocarbons, is due to homozygous deletion of the gene [86,87], GSTT1 weakness has been found to negatively impact on the metabolism of various potential carcinogens such as monohalomethanes, which are widely used as methylating agents, pesticides, and solvents [86,87]. Furthermore, GST cytosolic activity in olfactory epithelium, the highest among extrahepatic tissues [39,86], is of particular interest in MCS, where the role of odorous triggers is important. In fact, acetaldehyde is one of the most important chemicals that induce sick house syndrome and MCS [88]. As further examples of such behavior, PON1—a high density lipoprotein (HDL)-associated enzyme which reacts with toxic organophosphorus compound including insecticides (malathion) and nerve agents (sarin, somon, and diazinon) [89] by cleaving the homocysteine-thiolactone ring [40,90,91]—is known to be polymorphic in humans, with two isoforms displaying distinct hydrolyzing activities. The Arg192 isoform hydrolyzes paraoxon rapidly, whereas the Gln192 isoform acts slowly [92]. PON1 genes were associated with Gulf War Syndrome whose complaints of olfactory dysfunction are extensively shared with those of MCS [39]. On the other hand, the overlapping symptoms among MCS spectrum disorders have been found to possibly share some common pathogenetic features such as increased nitric oxide/peroxynitrite levels [57] and its consequences on the olfactory pathways [93]. In light of this, SOD2 genetic polymorphisms, which may be considered genetic determinants of MCS risk [40,42,43,44,52], seem to be connected to the loss of efficiency of detoxification systems, disturbances of free radical/antioxidant homeostasis, and increased production of inflammatory cytokines [31,48,94]. Interestingly, it has also recently been reported that gene variants of NOS2 are associated with the alteration of NO levels in inflammatory bowel disorders, asthma, atopy, olfactory dysfunction, and migraine [57,93,95,96], all of which are comorbidities shared by environmental sensitivity illnesses [57].

In this vision, enzymes also involved in xenobiotic metabolism phase I demonstrated an interface between detoxification function, olfactory perception, and neurodegenerative and psychiatric disorders. In fact, animal models showed that the inhibition of rat CYP P450 monooxygenases increased the electro-olfactogram response amplitude, suggesting a role for these enzymes in signal termination [97], and that an odorant metabolite resulting from the cytochrome-dependent metabolism was able to activate an olfactory receptor [98,99]. CYP2D6, which is present not only in liver, but also at lower levels in brain and other tissues [40,100], metabolizes a wide variety of substances including therapeutic drugs, drugs of abuse, procarcinogens, and neurotoxins [40], and is genetically polymorphic [101]. Thus, the 5–10% of Caucasians who are CYP2D6 homozygous for two non-functional alleles display impaired metabolism of many centrally acting drugs and toxins such as tricyclic antidepressants, selective serotonin re-uptake inhibitors, monoamine oxidase inhibitors, amphetamines, codeine, neuroleptics, and neurotoxins [40]. The enzyme also metabolizes endogenous neurotransmitters [102], which may be related to the observation that poor metabolizers score higher on scales of neuropsychological disorders [103]. Recent literature has provided evidence for a link between the CYP2D6 genotype and many neurodegenerative disorders [40,100,104]. Table 5 summarizes the potential mechanisms involving the above-mentioned genes, their activity, and the polymorphisms affecting their function, possibly underpinning the physiopathological processes of MCS.

However, it is not surprising that the multiple regression and the desirability model highlighted two environmental and anamnestic factors impacting on the LQrv score, worsening the quality of life level of olfactory dysfunction. In particular, the desirability model clearly showed (Table 4, Figure 2)—in line with the Pareto chart built on the regression analysis (Table 3, Figure 1)—that chemical compounds exposure and previous surgery events may accrue, so that the LQrv score may increase over the level of 28.67. Thus, this aspect tends to agree with the literature vision suggesting that a) environmental and medical-surgical exposures may increase the possibility of an over-sensitivity toward external compounds and odorants [31,57] and that b) this may be evidenced in those subjects in which the risk is increased due to a genetic predisposition [31,38,57].

In conclusion, the present work, which implemented for the first time a genetic-acquired factors model on a regression analysis, further reinforces those theories positing MCS as a complex, multifactorial disease in which the genetic risk related to defects in xenobiotic detoxification phase I and II enzymes also involved in olfactory and neurodegenerative diseases plays a necessary, and probably at all not sufficient role in the pathophysiological route for which the combination of multiple aspects including acquired/environmental determinants induce an over-sensitivity to olfactory compounds, finally impacting on quality of life.

## 4. Materials and Methods

### 4.1. Participants

We included in the study MCS patients admitted to the Lazio Regional Center for Diagnosis, Prevention and Treatment of MCS and evaluated at ‘‘Tor Vergata’’ University for those symptoms related to smell complaints. Diagnosis of MCS was achieved according to the US Consensus Criteria for MCS [105] and the revisions suggested by Lacour et al. [68,70,106], which were operationalized as follows: (1) symptoms present for at least six months; (2) symptoms occurred in response to exposure to at least two of 11 common volatile chemicals (many of which are reported in Table 6, according to previous studies [107]); (3) co-occurrence of at least one symptom from the CNS and one symptom from another organ system; (4) symptoms cause significant lifestyle changes; (5) symptoms occur when exposed and lessen or resolve when the symptom triggering agent is removed; and (6) symptoms triggered by exposure levels do not induce symptoms in other individuals who are exposed to the same levels. Diagnosis was supported by biochemical analyses, showing high levels of oxidative stress and inflammation (not reported here).

The Ethical Committee Board of IDI IRCCS (Rome, Italy) approved the protocol research (approval no. 121/CE/2008; approval date: 11/12/2008). The study adhered to the principles of the Declaration of Helsinki and all of the participants provided written informed consent after receiving a detailed explanation of the study.

### 4.2. Genotype Analysis

Eleven participants underwent genetic testing for the presence of functional polymorphisms in genes coding for detoxification phase I enzymes, namely CYP2C9, CYP2C19, and CYP2D6; detoxification phase II enzymes GSTP1, GSTM1, GSTT1, and UGT1A1; antioxidant enzymes SOD2, CAT, PON1, and NOS3, OGG1; immune response enzyme myeloperoxidase (MPO); and folate cycle/methylation enzyme 5,10-methylene-tetrahydrofolate reductase (MTHFR).

Genomic DNA (gDNA) was isolated from peripheral blood white cells using the PUREGENE-DNA purification system (GENTRA, QIAGEN, Milan), according to the manufacturer’s instructions. The gDNA was quantified by spectrophotometric measurement at 260 nm using a Biophotometer (Eppendorf). gDNA quality was considered acceptable for samples with a 260/280 ratio ≥ 1.6. DNA integrity and the presence of contaminant RNA was checked by electrophoresis on 0.8% agarose gel.

The screening for the presence of gene polymorphisms in the above cited genes was carried out by either real-time PCR-based allelic discrimination, direct DNA sequencing, and allele specific PCR.

#### 4.2.1. Allelic Discrimination by Real-Time PCR

The following gene polymorphisms were analyzed by real-time PCR using predesigned TaqMan Single Nucleotide Polymorphism (SNP) genotyping assays available from Applied Biosystems (ThermoFisher, Monza, Italy): CYP2C9*2 (C430T, rs1799853, R144C; assay ID: C__25625805_10), CYP2C9*3 (A1074C, rs1057910; assay ID: C__27104892_10), CYP2C19*2 (G681A, rs4244285; assay ID: C__25986767_70), CYP2D6*2 (2850C > T, R296C, rs16947; assay ID: C__27102425_10), CYP2D6*2A (-C1584G, rs1080985; C__32407252_30), CYP2D6*4 (G1846A, rs3892097; assay ID: C_27102431_D0), CYP2D6*41 (G2988A, rs28371725; assay ID: C__34816116_20), GSTP1 (A313G, I105V, rs1695; assay ID: C___3237198_20), SOD2 Ala16Val (C48T, rs4880; assay ID: C___1202883_20), CAT –C262T (rs1001179; C__11468118_10), NOS3 Glu298Asp (G894T, rs1799983; C___3219460_20), PON1 L55M (C108T, rs705379; assay ID: C__11708905_109), PON1 Q192R (A575G, rs662; assay ID: C___2548962_20), MTHFR C677T (Ala222Val, rs1801133; assay ID: C___1202883_20), and MTHFR A1298C (Glu429Ala, rs1801131; assay ID: C___850486_20). PCR conditions used were those previously reported [43,44,108,109,110]. PCR reactions were run in a Fast Real-time PCR 7900 (Applied Biosystems, ThermoFisher, Monza, Italy).

#### 4.2.2. Sanger Sequencing

The presence of gene polymorphisms CYP2C19*17, CYP2D6*10, MPO -G463A (rs2333227), OGG1 C315G (rs1052133, Ser326Cys), and UGT1A1*28 (-54-53insTA, rs8175347) was screened by DNA direct sequencing by Sanger methods.

Briefly, PCR reactions were performed in a total volume of 50 μL, containing 100 ng of genomic DNA, 10 × PCR buffer (20 mM Tris-HCl, pH 8.4; 50 mM KCl), 2.5 mM MgCl_2_, 25 pmoles of each primer, 0.1 mM dNTPs, 0.1 % Triton-X; and 2.5 U of Taq polymerase (EuroClone, Milan, Italy). Primer sequences used in this work were: OGG1 C315G were, respectively: CYP2C19*17 5′- AAGAAGCCTTAGTTTCTCAAG-3′ (*fwd*)/5′-AAACACCTTTACCATTTAACCC-3′ (*rev*); CYP2D6*10 5′- TCAACACAGCAGGTTCA -3′(*fwd*)/ 5′- CTGTGGTTTCACCCACC -3′(*rev*); UGT1A1*28 5′- GATTTGAGTATGAAATTCCAGCCAG -3′ (*fwd*)/5′- CCAGTGGCTGCCATCCACT -3′ (*rev*); MPO -G463A 5′- CGGTATAGGCACACAATGGTGAG -3′ (*fwd*)/5′- GCAATGGTTCAAGCGATTC -3′ (*rev*); OGG1 C315G 5′- GGAAGGTGCTTGGGGAAT -3′ (*fwd*)/ 5′- ACTGTCACTAGTCTCACCAG -3′ (*rev*). Primer sequences were designed by Primer 3 Input v. 0.4.0 software freely available online, except for those of UGTA1, which were derived by Massacesi et al. [111].

The PCR reactions were carried out in an Eppendorf Master Cycler Pro^®^ (Vapo-protect) PCR instrument (Eppendorf, Germany). After 35 cycles of amplification (denaturation at 94 °C for 30 s, annealing at 60 °C for 1 min, and extension at 72 °C for 1 min), the amplification products were electrophoresed in 2% agarose gel, and visualized after staining with ethidium bromide.

PCR products were purified as previously reported [112]. DNA sequencing of PCR products for the MPO gene (289 bp), OGG1 gene (189 bp), and UGT1A1 gene (351 bp) was performed using the Sanger method employing the BigDye Terminator v1.1 Cycle Sequencing kit (Applied Biosystems, Life Technologies, Milan, Italy). The reaction was carried out in a final volume of 20 µL, containing 25 ng of an appropriate amount (20 ng/100 bp) of purified PCR product, 5 pmol of the relevant primer, 2.5_Ready Reaction mix, 1_Sequencing Buffer, and DNAse/RNAse-free water, in the Eppendorf Master Cycler Pro^®^ (Vapo-protect) PCR instrument (Eppendorf, Germany). The thermal cycling conditions were: 96 °C for 30 s and 28 cycles, 30 s at 96 °C, 10 s at 50 °C, 4 min at 60 °C, and finally 5 min at 4 °C. The cycle sequencing products were purified and analyzed on an automated ABI PRISMs 310 Genetic Analyzer (Applied Biosystems, Applera Corp., Milan, Italy) as previously reported [112].

Genotyping calls were manually inspected and verified prior to release. MPO and OGG1 mutated genotypes were assigned on the basis of single nucleotide substitutions, when present. UGT1A1 genotypes were assigned based on the number of TA repeats for each allele (i.e., 6/6, 6/7, 7/7, or 7/8).

#### 4.2.3. Multiplex PCR Analysis for Deletion Polymorphisms

The deletion polymorphisms for the GSTM1 and the GSTT1 genes were determined simultaneously in a single assay using a multiplex PCR approach with the amplification of the GSTM1 and the GSTT1 genes from genomic DNA and using β-globin as the internal control, as previously described [44].

### 4.3. Olfactory Study

A detailed case history investigating events beyond the education level and occupation of chemical compounds exposure, physical and psychological trauma, and previous surgery was performed in all MCS subjects who underwent ear–nose–throat examination with fiberoptic check of the upper airways.

For the study of olfactory complaints during daily activities, we used the “life quality statements” from QOD that expressed the patients’ complaints related to the smelling difficulties [1,12,113]. There were in total nineteen life quality statements: seventeen negative statements (QOD-NS) with 3 points assigned for checking the box “agree”, 2 points for “partly agree”, 1 point for “partly disagree”, and 0 points for “disagree”, and two positive statements (QOD-PS) with 0 points by checking the box “agree”, 1 point “partly agree”, 2 points “partly disagree” and 3 points “disagree”. The sum of the scores for the QOD-NS and QOD-PS produces the LQ raw score (LQrv); a maximum of 57 points can be reached. High scores indicate a strong impairment [113].

Subjects with diabetes, oncologic or HIV history, neurological, and psychiatric or mood disorders, radiation, and traumatic brain injury were excluded from the study. No patient showed liver or renal abnormalities nor was pregnant or breastfeeding. Neurological diseases were excluded with the mini mental state examination and magnetic resonance imaging (MRI). All conditions that could potentially develop an olfactory dysfunction were considered as exclusion criteria. Thus, patients with sino-nasal disorders; neuro-psychiatric disorders (Parkinson’s disease, Alzheimer’s disease, schizophrenia, multiple sclerosis, and depression); lower airways and/or lung diseases; active hepatitis, cirrhosis, chronic renal failure, vitamin B12 deficiency; alcohol, tobacco, or drug abuse; cerebral vascular accidents; insulin dependent diabetes mellitus; hypothyroidism; and Cushing syndrome were not included in the study [114].

### 4.4. Data Handling and Statistical Analysis

In order to carry out statistical analyses, all selected polymorphisms were sub-grouped according to their functional effects and the enzyme role [71,115]. The following three classes were obtained: MTHFR (including MTHFR C677T and MTHFR A1298C), phase I (including CYP2D6, CYP2C19, UGT1A1*28, CYP2C9 A1075T, and CYP2C9 C430T), and phase II (including MPO G463A, GSTP1 A313G, GSTM1, GSTT1, SOD2 RS4880, CAT C262T, OGG1 C315G, PON1 A575G, PON1 C108T, and eNOSAsp298Glu). Wild-type, heterozygote, and mutated homozygote genotypes of the MTHFR C677T, MTHFR A1298C, MPO G463A, GSTP1 A313G, SOD2 RS4880, CAT C262T, OGG1 C315G, PON1 A575G, PON1 C108T, eNOSAsp298Glu, UGT1A1*28, CYP2C9 A1075T, and CYP2C9 C430T were vectorially scored from 0 to 2, respectively. Wild-type and deleted genotype of both GSTM1 and GSTT1 were scored 0 and 1, respectively, and the ultra-rapid, extensive, intermediate and slow metabolizer phenotype of CYP2D6 and CYP2C19 was scored from −1 to 2. A composite score for each class was computed by adding the score of the single form of the enzymes. Absence and presence of events of chemical compounds exposure, physical and psychological trauma, and previous surgery were scored 0 and 1, respectively. Number and percentage of cases for each gene condition and clinical–anamnestic aspect was calculated and compared with the literature findings by means of the Fisher’s exact test. The X^2^ test was carried out to define associations between categorical factors and groups. To assess that data were of Gaussian distribution, D’Agostino K squared normality and Levene’s homoscedasticity test were applied (where the null hypothesis is that the data are normally and homogenously distributed) [116].

Given the exploratory nature of the study and previous biomedical approaches [117], correlations between the LQrv and nine prognostic factors (genotypic/phenotypic score of MTHFR, phase I and phase II classes, absence/presence of events of chemical compounds exposure, physical and psychological trauma and previous surgery, age, and gender (0 = male, 1 = female) were examined by a multiple regression analysis. P-values less than 0.05 were considered as statistically significant. A Pareto chart contrasting the *t*-values and *p*-values of each factor were generated. Finally, a two-sided desirability model was achieved for each prognostic factor, according to the mentioned regression model and setting upper and lower limit of LQrv at 22 and 40, representing the lowest and highest desirability values, respectively [117,118]. Subsequently, the partial desirability functions (d_i_) were combined into a single composite global desirability function, defined as the geometric mean of the different d_i_ values, implying that all responses are in a desirable range simultaneously and the combination of the different criteria is therefore globally optimum, so the response values are near the target values.

## 5. Limitations of the Study

The results of this study have to be considered with caution, because of their preliminary nature and considering the following limitations. In fact, no correction for multiple comparisons was performed in the multiple regression model. If this exploratory—rather than confirmatory—approach is extensively adopted in similar studies [117,119,120] to reduce the likelihood of Type II statistical errors as much as possible, this choice may have induced the increase in likelihood of Type I statistical errors. However, given these assumptions, the present data have to be considered as preliminary and should be replicated in further cohorts of patients.

## Figures and Tables

**Table 1:** Olfactory-related quality of life and clinical-anamnestic aspects of Multiple Chemical Sensitivity patients.

QOD NS	23.8 ± 4.56
QOD PS	4.86 ± 0.97
LQrv (NS + PS)	28.67 ± 4.22
Age (years)	47.23 ± 10.06
Gender	27 females; 19 males
Compounds exposure	24
Psychological trauma	17
Physical trauma	14
Previous surgery	14

Legend: Olfactory-related quality of life and clinical-anamnestic aspects in 46 MCS patients. Questionnaire of Olfactory Disorders, QOD; negative statements, NS; positive statements, PS; sum of the scores for the QOD-NS and QOD-PS = quality of life raw score, LQrv. Where needed means ± standards deviations are given.

**Table 2:** Distribution of gene polymorphisms of enzymes involved in xenobiotic metabolism, oxidative stress, and DNA methylation/repair pathways.

Gene Polymorphism	Genotype/Alleles	Genotype and Allele Frequency in MCS Patients	Genotype and Allele Frequency in Caucasian Population	*p*
CYP2C9 C430T, *2 (R144C) A1075T, *3 (I359L)	CC	63.0%	80% ^§1^	0.0273
	CT	34.8%	17.2% ^§1^	0.0044
	TT	2.2%	2.8% ^§1^	0.9833
	C(*1), T(*2)	0.804, 0.196	0.886, 0.124 ^§1^	
	AA	82.6%	98.9% ^§1^	<0.00001
	AT	17.4%	1.1% ^§1^	<0.00001
	TT	0%	0% ^§1^	-
	A(*1), T(*3)	0.913, 0.087	0.994, 0.056 ^§1^	
	*2/*3	10.9%	2.2% ^§1^	0.0097
CYP2C19 G681A, *2 -C806T, *17	*1/*1	43.5%	49.2% ^§2^	0.252
	*1/*2	21.7%	16.4% ^§2^	0.279
	*2/*2	2.2%	2.8% ^§2^	1
	*1/*17	30.4%	22.8% ^§2^	0.179
	*17/*17	2.2%	2.8 ^§2^	1
	*1, *2, *17	0.695, 0.130, 0.175	0.688, 0.110, 0.142 ^§2^	
CYP2D6 C2850T, *2 (R296C) -C1584G, *2A G1846A, *4 1707delT, *6 C100T, *10 G2988A, *41	PM ^#^ (4*10/4*10)	6.5%	6.7% ^§2^	1.0
	IM ^#^ (*1/*4/*10, *2*2A/*4*10 *10/*4*10)	6.5%	28.9% ^§2^	0.006
	EM ^#^ (1*/*1, *2/*2, *2A/*2A, *2*41/*2*2A *2*4*10/*2A, *4/*10, *1/*6)	80.5%	62.2% ^§2^	0.154
	UM ^#^ (*2/*2A*XN)	6.5%	2.2% ^§2^	0.617
GSTP1 A313G	AA	41.3%	23.5% ^§4^	0.0074
	AG	50.0%	62.5% ^§4^	0.0943
	GG	8.7%	14% ^§4^	0.4888
	A, G	0.663, 0.337	0.70, 0.30 ^§4^	0.4573
GSTM1 DEL	INS, DEL	0.413, 0.587	0.527, 0.473, ^§5^	0.4573
GSTT1 DEL	INS, DEL	0.761, 0.239	0.78, 0.22 ^§5^	0.8263
GSTM1/GSTT1	INS/DEL	15%	15% ^§3^	0.96
GSTM1/GSTT1	DEL/INS	50.3%	41% ^§3^	0.25
GSTM1/GSTT1	DEL/DEL	9.0%	1.5% ^§3^	0.85
**UGT1A1** (TA)_7_TAA, *28	*1/*1	32.6%	48.5% ^§6^	0.0532
	*1/*28	52.2%	39.0% ^§6^	0.135
	*28/*28	15.2%	12.5% ^§6^	0.631
	*1, *28	0.587, 0.413	0.701, 0.299 ^§6^	
**Gene Polymorphism (Amino Acid Substitution)**	**Genotype/Alleles**	**Genotype and Allele Frequency in MCS Patients**	**Genotype and Allele Frequency in Caucasian Population**	***p***
SOD2 C48T (A16V)	CC	19.6%	40.2% ^§7^	0.0076
	CT	47.8%	45.2% ^§7^	0.749
	TT	32.6%	14.6% ^§7^	0.0056
	C, T	0.435, 0.565	0.628, 0.372 ^§7^	
CAT -C262T	CC	56.5%	80% ^§8^	0.0008
	CT	32.6%	19% ^§8^	0.0415
	TT	10.9%	1% ^§8^	0.0004
	C, T	0.728, 0.272	0.895, 0.105 ^§8^	
PON1 A575G (Q192R) C108T (L55M)	AA	50%	52.1% ^§9^	0.4485
	AG	39.1%	36.3% ^§9^	0.7202
	GG	10.9%	7.6% ^§9^	0.4551
	A, G	0.696, 0.304	0.742, 0.258 ^§9^	
	CC	28.3%	19.9% ^§4^	0.3868
	CT	47.8%	54.4% ^§4^	0.0002
	TT	23.9%	26.7% ^§4^	0.014
	C, T	0.522, 0.478	0.461, 0.539 ^§4^	
	AG/CT	10.9%	/	
NOS3 G894T (D298E)	GG	45.7%	36.9% ^§10^	0.3093
	GT	32.6%	47.8% ^§10^	0.0814
	TT	21.7%	15.3% ^§10^	0.3316
	G, T	0.619, 0.381	0.608, 0.392 ^§10^	
MPO G463A	GG	43.5%	64.9 ^§11^	0.057
	GA	45.6%	35% ^§11^	0.1535
	AA	10.9%	0.1% ^§11^	0.4436
	G, A	0.337, 0.663	0.76, 0.24 ^§11^	
MTHFR C677T (A222V) A1298C (E429A)	CC	34.8%	37.6% ^§12^	0.7188
	CT	43.5%	48.5% ^§12^	0.3766
	TT	23.9%	13.9% ^§12^	0.091
	C, T	0.543, 0.457	0.619, 0.381 ^§12^	
	AA	41.3%	51% ^§12^	0.256
	AC	47.8%	39.6% ^§12^	0.323
	CC	10.9%	9.4% ^§12^	0.783
	A, C	0.772, 0.228	0.708, 0.292 ^§12^	
	CT/AC	23.9%	18.4%^§4^	0.505
OGG1 C315G (S326C)	CC	78.3%	65.0% ^§13^	0.094
	CG	19.6%	32.2% ^§13^	0.0893
	GG	2.2%	2.8% ^§13^	1
	C, G	0.804, 0.196	0.811, 0.189 ^§13^	

Legend: ^§1^ Serpe et al., 2015 [58]; ^§2^ Martis et al., 2013 [59]; ^§^3 Serrano et al., 2011 [60]; ^§4^Antognelli et al., 2009 [53]; ^§5^ Boccia et al., 2007 [61]; ^§6^ Chen et al., 2015 [62]; ^§7^ Palmirotta et al., 2015 [55]; ^§8^ Malinowska et al., 2016 [56]; ^§9^ Tetik Vardarli et al., 2017 [63]; ^§10^ Zakrzewski-Jakubiak et al., 2008 [64]; ^§11^ Roszak et al., 2016 [65]; ^§12^ Mazzuca et al., 2015 [66]; ^§13^ Moreno et al., 2006 [67]. ^#^ PM = Poor Metabolizer, IM = Intermediate Metabolizer, EM = Extensive Metabolizer, UM = Ultrametabolizer.

**Table 3:** Multiple regression model of the olfactory-related life quality (LQrv) in relation to genetic and clinical-anamnestic factors.

	Partial Regression Coefficient	Std.Err	t	*p*-Value	Cnf.Lmt −95.00%	Cnf.Lmt +95.00%	Partial Correlation Coefficient (ß)	Std.Err. ß	Cnf.Lmt −95.00%	Cnf.Lmt +95.00%
Intercept	18.63568	1.953092	9.541631	**0.000000**	14.67463	22.59673				
Phase I enzymes score	1.09827	0.316817	3.466581	**0.001382**	0.45574	1.74081	0.288895	0.083337	0.119879	0.457910
Compounds Exposure	2.53639	0.976863	2.596462	**0.013552**	0.55522	4.51756	0.303415	0.116857	0.066418	0.540412
Previous Surgery	1.89423	0.812950	2.330064	**0.025525**	0.24549	3.54296	0.208725	0.089579	0.027050	0.390400
Phase II enzymes score	0.40192	0.174334	2.305473	**0.027012**	0.04836	0.75549	0.235737	0.102251	0.028362	0.443113
MTHFR enzymes score	0.87996	0.454025	1.938130	0.060481	−0.04085	1.80076	0.176058	0.090839	−0.008172	0.360289
Psychological Trauma	1.25605	0.999760	1.256353	0.217080	−0.77156	3.28366	0.150255	0.119596	−0.092297	0.392807
Age	0.04117	0.034592	1.190050	0.241816	−0.02899	0.11132	0.098115	0.082446	−0.069093	0.265323
Gender	0.81441	0.777464	1.047519	0.301843	−0.76236	2.39118	0.096030	0.091673	−0.089893	0.281952
Physical Trauma	−0.28221	0.952656	−0.296230	0.768757	−2.21428	1.64987	−0.032621	0.110119	−0.255953	0.190712

Legend: Table depicting the multiple regression model of the olfactory-related life quality (LQrv) in relation to genetic and clinical-anamnestic factors. MTHFR, folate cycle/methylation enzyme; Std., standard; Err, error; Cnf., confidence; Lmt, limit. In bold significant *p*-values.

**Table 4:** Partial desirability model of main factors predicting olfactory-related quality of life in MCS patients.

Partial Desirability Values					
Prognostic Factor	Factor Level	Predicted LQrv	Desirability Value	−95% CI LQrv	+95% CI LQrv
MTHFR enzymes score	−0.363323	27.18730	0.388626	25.49463	28.87997
	0.4813819	27.93061	0.444314	26.90583	28.95539
	1.326087	28.67391	0.500001	28.00669	29.34114
	2.170792	29.41722	0.532814	28.39244	30.44200
	3.015497	30.16052	0.565628	28.46785	31.85319
Phase II enzymes score	0.8952978	26.68338	0.350873	24.80952	28.55724
	3.371562	27.67865	0.425437	26.57786	28.77943
	5.847826	28.67391	0.500001	28.00669	29.34114
	8.324090	29.66918	0.543937	28.56839	30.76996
	10.80035	30.66444	0.587874	28.79059	32.53830
Phase I enzymes score	−0.721111	26.23453	0.317246	24.65912	27.80994
	0.3894446	27.45422	0.408623	26.47730	28.43114
	1.500000	28.67391	0.500001	28.00669	29.34114
	2.610555	29.89360	0.553845	28.91669	30.87052
	3.721111	31.11329	0.607689	29.53789	32.68870
Compounds exposures	−0.488355	26.11192	0.308060	24.00246	28.22139
	0.0166921	27.39292	0.404031	26.19027	28.59556
	0.5217391	28.67391	0.500001	28.00669	29.34114
	1.026786	29.95491	0.556551	28.75226	31.15755
	1.531833	31.23590	0.613102	29.12643	33.34537
Psychological trauma	−0.488355	27.40518	0.404949	25.25116	29.55920
	0.0166921	28.03955	0.452475	26.81732	29.26178
	0.5217391	28.67391	0.500001	28.00669	29.34114
	1.026786	29.30828	0.528005	28.08605	30.53051
	1.531833	29.94264	0.556010	27.78862	32.09666
Physical Trauma	−0.606476	28.94936	0.512160	26.94901	30.94970
	−0.118456	28.81164	0.506080	27.65654	29.96673
	0.3695652	28.67391	0.500001	28.00669	29.34114
	0.8575860	28.53619	0.489683	27.38110	29.69128
	1.345607	28.39847	0.479365	26.39813	30.39881
Previous Surgery	−0.626082	26.91147	0.367961	25.23861	28.58433
	−0.160867	27.79269	0.433981	26.77608	28.80931
	0.3043478	28.67391	0.500001	28.00669	29.34114
	0.7695630	29.55514	0.538903	28.53852	30.57175
	1.234778	30.43636	0.577805	28.76350	32.10922
Age	27.11425	27.84545	0.437933	26.28385	29.40705
	37.17669	28.25968	0.468967	27.28832	29.23104
	47.23913	28.67391	0.500001	28.00669	29.34114
	57.30157	29.08815	0.518287	28.11679	30.05950
	67.36401	29.50238	0.536574	27.94078	31.06398
Gender	−0.408686	27.86305	0.439252	26.15725	29.56886
	0.0891352	28.26848	0.469627	27.23827	29.29869
	0.5869565	28.67391	0.500001	28.00669	29.34114
	1.084778	29.07934	0.517899	28.04913	30.10955
	1.582599	29.48477	0.535797	27.77897	31.19058

Legend: Partial desirability model depicting in MCS subjects the predicted values of main genetic and clinical-anamnestic factors when contrasted against olfactory-related quality of life (LQrv). MTHFR, folate cycle/methylation enzyme; CI, confidence interval.

**Table 5:** Metabolic role of enzymes for which the occurrence of gene variants, affecting enzyme activity, has been observed in MCS patients.

Enzyme Name	Gene Symbol	Catalyzed Reaction	Description of Enzyme Activity	Gene Polymorphisms Affecting Enzyme Function *
*Catalase*	CAT	2 H_2_O_2_ ⇄ 2 H_2_O + O_2_	Decomposition of hydrogen peroxide to water and oxygen, protecting the cells from oxidative damage by ROS. Catalase reactions are a key part of body’s enzymatic antioxidant defense mechanisms.	CAT-262C > T
*Cytochrome P450 monoxygenase isozymes* *2C9, 2C19, 2D6*	CYP2C9 CYP2C19 CYP2D6	RH + O_2_ + NADPH + H^+^ → ROH + H_2_O + NADP^+^	Insertion of one atom of oxygen (monoxygenation) into the aliphatic position of an organic substrate (RH), while the other oxygen atom is reduced to water. This biotransformation reaction is often referred to as “phase I detoxification” in xenobiotic metabolism.	CYP2C9 *2, *3 CYP2C19 *2, *17 CYP2D6 *4, *6, *10, *41
*Glutathione-S-transferase isozymes P1, M1, T1*	GSTP1 GSTM1 GSTT1	GSH + X → GS-X + H^+^	Conjugation of reduced glutathione (GSH) with toxic agents, carcinogens, drugs, leading to the formation of a detoxified complex more polar and more readily excreted from human body. This reaction is often referred to as “phase II detoxification” in xenobiotic metabolism.	GSTP1 I105V, A114V, GSTM1 null GSTT1 null
*Methylenetetrahydrofolate reductase*	MTHFR	5,10-MTHF + NADPH → 5-MTHF+ NADP^+^	Reduction of 5,10-methylenetetrahydrofolate (5,10-MTHF) to 5-methyltetrahydrofolate (5-MTHF), acting as methyl donor for homocysteine (Hcy) remethylation to methionine. Enzyme activity diminishment leads to Hcy accumulation that induces oxidative stress and endothelial dysfunction.	MTHFR C677T (A222V) MTHFR A1298C (E429V)
*Myeloperoxidase*	MPO	H_2_O_2_ + X^−^ ⇄ H_2_O + HOX	MPO-mediated reaction of hydrogen peroxide with halide anions (X-),chloride, bromide, fluoride, iodide) generates hypohalous acids, that mediate the anti-microbial activity of neutrophil granulocytes, key cells of immune system.	MPO-G463A
*Nitric oxide synthase type III*	NOS3	2 L-Arginine + 3 NADPH + 3 H^+^ + 4 O_2_ ⇄ 2 Citrulline + 2 NO + 4 H_2_O + 3 NADP^+^	Production of nitric oxide (NO) from L-arginine in the presence of NADPH, as cofactor, and oxygen. NO is a potent mediator of vasodilation in blood vessels.	NOS3 G894T (D298E)
*8-Oxoguanine glycosylase*	OGG1	8-oxoG excision → nucleotide gap in DNA sequence	Excision of 8-oxoguanine (8-oxoG), an oxidized deoxyribonucleotide, with mutagenic effects, resulting from DNA exposure to ROS. OGG1-mediated cleavage of glycosidic bond causes a strand break in the DNA backbone.	OGG1 C315G (S326C)
*Paraoxonase 1*	PON1	OP + H_2_O → DEP + Phenol-X	Hydrolysis of pesticides organophosphates (OP) to diethylphosphate (DEP) and phenol compounds (Phenol-X: PNP, IMHP, TCP). PON1, as a HDL component, also protects against atherosclerosis by preventing the oxidation of plasma lipoproteins and the accumulation of oxidized LDLs and HDLs.	PON1 C108T (L55M), PON1 A575G (Q192R)
*Superoxide dismutase 2*	SOD2	2O_2_^−^ + 2H^+^ ⇄ O_2_ + H_2_O_2_	Dismutation of superoxide radicals (O_2_^−^) to molecular oxygen (O_2_) and hydrogen peroxide (H_2_O_2_). SOD2 is a manganese-dependent enzyme located in mitochondria, providing to human cells a greatly effective defense against ROS.	SOD2 C28T (A16V)
*UDP-glucuronosyl transferase isozyme 1A1*	UGT1A1	UDP-GA + X → UDP + GA-X	Transfer of the glucuronic acid component of UDP-glucuronic acid (UDP-GA) to a small hydrophobic molecule (X) in microsomal compartment. *Glucuronidation* reaction takes place in phase II detoxification of xenobiotic metabolism.	UGT1A1 (TA)_7_TAA, * 28

Legend: HDL, high density lipoprotein; LDL, low density lipoproteins; OP, organophosphates pesticides and insecticides such as paraoxon, diazoxon, and chlorpyrifos; Phenol-X: phenolic compounds, such as p-nitrophenol, isopropyl methyl pyrimidinol, and trichloropyridinol; X, xenobiotics, toxic, carcinogens, drugs. * Polymorphisms examined in this study.

**Table 6:** List of odorants and respective chemical names.

Odorants	Chemical Names	Odorants	Chemical Names
Almond	*Benzaldehyde*	Lavender	*Essential oil*
Anise	*Anethol*	Lemon	*Citral*
Apple	*Ethyl 2-methylbutyrate*	Mint	*Essential oil*
Banana	*Isoamyl acetate*	Mushroom	*1-Octen-3-one*
Bell pepper	*2-Isobutyl-3-methoxypyrazine*	Musk	*Omega-pentadecalactone*
Butter	*Diacetyl*	Onion	*Ethanethiol*
Cabbage	*Methionol*	Orange blossom	*Methyl anthranilate*
Camembert	*S-methylthiobutyrate*	Peach	*γ-Undecalactone*
Caramel	*Furaneol*	Pear	*Hexyl acetate*
Cinnamon	*Cinnamaldehyde*	Pineapple	*Allyl hexanoate*
Cloves	*Eugenol*	Plastic	*Styrene*
Coconut	*Whiskey-lactone*	Rose	*Phenylethanol*
Coffee	*Extract*	Rubber	*Benzothiazole*
Coriander	*Linalool*	Sea	*Calone*
Cork taint	*2,4,6-Trichloroanisole*	Smokey	*4-Ethylgaïacol*
Crab stick	*Dimethylsulfide*	Sweat	*3-Sulfanylhexyle acetate*
Earthy	*(±)-Geosmin*	Thyme	*Thymol*
Eucalyptus	*1,8-Cineol*	Toasted bread	*2-Acetylthiazole*
Feet	*Isovaleric acid*	Vanilla	*Aroma*
Fish	*2-Butylamine*	Vinegar	*White vinegar*
Grass	*Cis-3-hexenol*	Violet	*β-Ionone*
Horse	*4-Ethylphenol*	Washing powder	*Aldehyde*
Kiwi	*Ethyl butyrate*	Woody	*Cedryl acetate*

Legend: List of common odorants (with grey background) and their chemical names (in italics).
